# Prevalence and impact of S*treptococcus pneumoniae* in adult cystic fibrosis patients: a retrospective chart review and capsular serotyping study

**DOI:** 10.1186/s12890-015-0041-z

**Published:** 2015-05-02

**Authors:** Christina S Thornton, Erin L Brown, Joenel Alcantara, Harvey R Rabin, Michael D Parkins

**Affiliations:** Department of Microbiology, Immunology and Infectious Diseases, Calgary, Canada; Department of Medicine, Adult Cystic Fibrosis Clinic, Calgary, Canada

**Keywords:** Pneumococcus, Eradication, Pulmonary exacerbation, Pneumonia

## Abstract

**Background:**

Cystic fibrosis (CF) is a genetic disease characterized by complex polymicrobial communities within the lower respiratory tract. *S. pneumoniae,* while a well-defined pathogen in the general population, has rarely been identified in CF. Furthermore, prevalence studies on Pneumococcus in CF have predominantly focused on the infant and pediatric populations, and outcome data is lacking.

**Methods:**

Through a review of our comprehensive clinical and microbiologic database from a single adult CF center in Canada from 1978–2013 we sought to determine the incidence, prevalence, serotype and clinical impact of Pneumococcus in adults with CF.

**Results:**

Only fifteen of 318 adult CF patients (5%) were ever found to have transient Pneumococcus colonization, and none developed persistent infection although length of carriage varied. As all isolates were stored, capsular serotyping could be performed using a multiplex PCR panel. Capsular serotyping revealed a varied distribution of several serotypes within these isolates. Lung function testing at time of incident Pneumococcus isolation was compared with values before and after isolation and showed no significant reduction in spirometry values, nor was there an increased need for rescue antibacterial therapy.

**Conclusion:**

Within our center, incident Pneumococcus infection is neither common, associated with a disproportionate clinical deterioration nor results in chronic infection.

## Background

*Streptococcus pneumoniae* is an important respiratory pathogen that has been implicated in several respiratory diseases including otitis media, pneumonia and sinusitis [1]. The prevalence of pneumococcal nasal-pharyngeal asymptomatic colonization in the healthy adult population has been estimated to be at 20-50% [2]. Isolation of Pneumococcus in CF has been predominantly studied in infants and young children [1,3]. Several studies have addressed the pathogenic potential of Pneumococcus in CF and have found increased biofilm formation, increased antibiotic resistance [4] and the isolation of this species during acute exacerbation [1]. Studies on prevalence within adult CF patients are limited; with one group finding ten percent of CF patients had cultivatable isolates from routine sputum samples during periods of clinical stability when specifically sought using enhanced culture techniques [2]. The impact of isolating Pneumococcus from sputum on short and long term CF disease progression, however, is unknown.

The objectives of this study were to do a retrospective longitudinal study of adult CF patients to assess Pneumococcus incidence and prevalence as determined by routine culture practices. Clinical impact of infection was evaluated by comparing requirement for antibacterial therapy and changes in lung function before and after infection. Finally, phenotypic characterization was performed including antibiotic susceptibility and capsular serotypes of the pneumococci isolates to assess distribution within this cohort of adult CF patients.

## Methods

A retrospective audit was performed on the Adult Cystic Fibrosis Clinic of Southern Alberta, Canada’s comprehensive sputum biobank. This prospectively collected and maintained biobank includes frozen stock of every bacterial isolate ever identified, from every clinical encounter between 1978–2013. Inclusion of patients into this study was dependent on a diagnosis of CF, and the confirmation of positive culture(s) of *S. pneumoniae* from any sputum sample done routinely at quarterly clinic visits and during periods of exacerbation. Corresponding clinical information was collected through detailed chart review including baseline demographic details, and the clinical course before and following isolation of Pneumococcus. Differences for percent predicted FEV_1_ (forced expiratory volume in one second) and FVC (functional vital capacity) lung function tests were assessed using the Student’s unpaired *t*-test. This study complies with the University of Calgary Conjoint Health Research Ethics Board (CHREB E-23087).

Sputum samples collected in real time were mixed with equal volumes of dithiothreitol (DTT, Sputolysin, EMD Millipore Billerica, MA) and vigorously vortexed. Samples were then serial diluted in DTT to a final dilution of 10^−5^. Samples diluted at 10^−3^ and 10^−5^ were then plated on routine CF media including: Columbia Blood Agar (CBA), MacConkey (MAC), Chocolate Agar (CHOC), Mannitol Salt Agar (MSA) and in the later years Burkholderia cepacia agar. *S. pneumoniae* were identified through standard approaches including flat, centrally depressed alpha-hemolytic colonies on CBA/CHOC, which were Gram positive diplococcoid, catalase-negative, Optochin sensitive (generally) and bile salt soluble. Isolates were frozen in skim milk stocks and maintained indefinitely at −80°C [5,6].

In 2014, *S. pneumoniae* isolates cataloged at any time point from the biobank were then targeted and subbed out from frozen stock and incubated in 5% CO_2_ at 37°C for 48–72 hours and their identity re-confirmed. DNA was extracted from isolates using boiling preparation where colonies were re-suspended in 50 ul of ddH_2_0, boiled for 15 minutes and then centrifuged at 13,000 rpm for 10 minutes. Capsular serotyping PCR was performed following the protocol described by Jourdain, *et al.* to screen for 30 classical *S. pneumoniae* colonizing serotypes [7]. A positive control of genomic DNA from *S. pneumoniae* strain ATCC 6303 was used alongside the internal *cps* loci within the multiplex PCR for comparison. Each PCR reaction was run on two separate occasions to ensure accuracy. Susceptibility testing was performed in real time to penicillin and those samples recovered had susceptibility testing to other common anti-Pneumococcal agents as well as those antibiotics commonly used in CF, using the Kirby-Bauer method as per the Clinical and Laboratory Standards Institute (CLSI) [8].

## Results

During the 34 years assessed only 27 *S. pneumoniae* isolates were identified from the 33,166 (0.08%) bacterial isolates collected from expectorated sputum samples from 318 patients in our biobank (Table 1). All isolates were cultured from sputum in real time at colony forming units (CFU) ranging from 10^4^-10^7^/ml (Table 2). No invasive isolates were identified. These were from 15 patients; 10 males and 5 females with a median age of 28.0 (IQR (inter-quartile range) 23, 30) years at time of first isolation. 11/15 of the patients were pancreatic insufficient and 67% 10/15 also had a major CF co-morbidity. Co-morbidities included CF related diabetes (3/15), arthropathy (3/15), liver and biliary problems (12/15) and sinonasal manifestations (5/15). Interestingly, one-third of the patients were chronic smokers. The median (IQR) for baseline FEV_1_ was 75.5% predicted (62.5, 105.5) and for FVC was 82.6% predicted (71.5, 112.5). Baseline chronic microbial pathogens included *P. aeruginosa* (12/15)*, S. aureus* (7/12)*, H. influenzae* (4/15) and *Burkholderia cepacia* complex (2/15). 8/15 of the patients had co-infection with two or more of the principal pathogens listed above. None of the samples were from patients who had previously undergone lung transplantation.

**Table 1:** **Adult cystic fibrosis patient demographics from those with isolated Pneumococcus cultures**

**Patient**	**Age** ^**a**^	**Sex**	**Mutation 1**	**Mutation 2**	**BMI** ^**b**^	**FVC (L)** ^**c**^ **(% predicted)**	**FEV** ^**1**^ **(L)** ^**d**^ **(% predicted)**	**Pancreatic sufficient**	**Smoker status**	**CF Co-morbidities**	**Chronic pathogens** ^**e**^
1	30	M	M1101K	M1101K	21.87	1.62 (35)	0.76 (20)	N	N	N	PA, SA, HI, Bcc
2	23	M	F508del	M1101K	16.05	3.29 (61)	0.94 (20)	N	N	Liver disease	PA, SA
3	30	F	F508del	P67L	32.03	N/A	N/A	Y	Y	N	SA
4	28	M	F508del	F508del	18.23	3.75 (68)	2.42 (54)	N	N	Sinus	PA, Bcc
5	29	F	F508del	F508del	15.58	2.06 (53)	1.08 (32)	N	N	CFRD, Sinus	PA
6	24	M	F508del	D110H	24.38	7.57 (140)	5.98 (128)	Y	Y	N	HI
7	24	M	F508del	F508del	16.20	3.77 (73)	1.4 (32)	Y	N	Sinus	PA
8	30	M	3659delC	394delTT	18.79	3.95 (75)	1.80 (41)	N	Y	Liver disease	PA
9	28	M	F508del	F508del	21.20	6.1 (117)	4.3 (100)	N	N	N	PA, SA
10	23	M	F508del	G551D	20.1	5.08 (88)	3.55 (74)	N	N	Sinus	PA, SA
11	30	F	F508del	Unknown	19.0	1.58 (35)	1.08 (29)	N	N	CFRD	PA
12	18	M	F508del	G178R	20.8	4.04 (107)	3.01 (86)	N	Y	Arthropathy, Sinus	SA, HI
13	28	F	F508del	F508del	19.7	2.48 (73)	1.5 (51)	N	N	N	PA
14	40	F	F508del	F508del	25.4	4.45 (134)	3.08 (110)	N	Y	CFRD, Arthropathy	PA, HI
15	22	M	F508del	F508del	21.7	4.26 (85)	2.94 (68)	N	N	Arthropathy	PA, SA

^a^Age at time of first isolation of *S. pneumoniae*.

^b^BMI: body mass index.

^c^FVC: forced volume capacity, defined as an average of two values in the year preceding time of pneumococcus isolation.

^d^FEV_1_: forced expiratory volume, defined as an average of two values in the year preceding time of pneumococcus isolation.

^e^PA: *P. aeruginosa,* SA: *S. aureus,* HI: *Haemophilus influenzae,* Bcc: *Burkholderia cepacia* complex; chronicity as defined by repeated isolation of pathogen on at least 50% of cultures over the prior two years.

**Table 2:** **Pneumococcus isolates from 1986–2013 in adult cystic fibrosis patients**

**I**	**Patient** ^**a**^	**Date of isolation**	**Capsular serotype** ^**b**^	**CFU**					**Influenza vaccine** ^**d**^	**Decreased PFT** ^**e**^	**Need for Anti-biotics** ^**f**^	**Co-Isolated organisms (CFU)** ^**g**^
					**Antibiotic susceptibility** ^**c**^							
					**PEN**	**LVX**	**AZM**	**CLI**				
1	1	1986-05-21	N/A^h^	10^7^	N/A	N/A	N/A	N/A	Y	N	Y	PA non-muc (10^7^), PA muc (10^5^) Bcc (10^6^), HI (10^5^)
2	2	1994-03-16	6A/B	10^7^	S	S	S	S	Y	Y	Y	PA non-muc (10^5^), PA muc (10^7^) SA (10^8^)
3	3	1992-09-18	N/A	10^7^	S	N/A	N/A	N/A	N	N/A	N	SA (10^8^)
4	3	1992-12-16	N/A	10^8^	S	N/A	N/A	N/A	Y	N/A	N	SA (10^8^), HI (10^8^)
5	3	1993-03-04	22	10^7^	S	S	S	S	N	N	Y	SA (10^7^), HI (10^8^)
6	3	1993-03-05	9A/V	10^7^	S	S	S	S	N	N	Y	SA (10^7^), HI (10^8^)
7	4	1993-03-17	6A/B	10^7^	S	S	S	S	Y	N	N	PA non-muc (10^7^), PA muc (10^7^), Bcc (10^5^), EC (10^5^)
8	5	1996-12-04	33A/F	10^7^	S	S	S	S	Y	N	N	PA non-muc (10^6^), PA muc (10^7^)
9	5	2009-04-08	23A	10^6^	S	S	S	S	Y	N	Y	PA muc (10^5^)
10	6	1990-01-17	N/A	10^7^	S	N/A	N/A	N/A	Y	N	N	CA (10^7^)
11	7	1993-01-06	N/A	10^7^	S	N/A	N/A	N/A	Y	N	N	PA non-muc (10^7^), PA muc (10^7^)
12	8	1995-10-18	23 F	10^7^	S	S	S	S	Y	Y	Y	PA non-muc (10^7^), PA muc (10^6^)
13	8	1995-11-01	23 F	10^7^	S	S	S	S	Y	Y	Y	PA non-muc (10^7^), PA muc (10^6^)
14	9	1996-10-02	38	10^7^	R		R	S	Y	Y	N	PA non-muc (10^6^), SA (10^6^), CA (10^5^), Asp (10^5^)
15	10	1995-12-20	7B/C	10^5^	S	S	S	S	Y	Y	N	PA muc (10^5^)
16	10	1996-07-31	14	10^4^	S	S	S	S	Y	N	N	PA muc (10^6^), SA (10^4^)
17	10	1996-08-28	9A/V	10^5^	S	R	R	S	Y	N	N	PA muc (10^7^), CA (10^4^)
18	11	1995-11-22	23B	10^7^	R	S	S	S	Y	N	Y	PA non-muc (10^6^), PA muc (10^7^), EC (10^5^)
19	11	1996-02-21	23B	10^7^	S	S	S	S	Y	N	Y	PA non-muc (10^7^), PA muc (10^7^), EC (10^6^)
20	12	2001-08-21	N/A	10^7^	S	N/A	N/A	N/A	N	N	Y	OF (10^7^)
21	12	2003-08-20	NT^i^	10^6^	S	R	R	S	Y	N	N	N/A
22	12	2006-07-17	N/A	10^6^	S	N/A	N/A	N/A	Y	N/A	N/A	N/A
23	12	2006-07-21	NT	10^6^	S	R	S	S	Y	N	N	Cfc (10^4^), HI (10^6^)
24	12	2009-01-05	14	10^5^	S	R	R	S	Y	N	N	HI (10^6^)
25	13	2004-06-16	N/A	10^7^	S	N/A	N/A	N/A	Y	N	N	PA muc (10^7^)
26	14	2003-11-26	N/A	10^6^	S	N/A	N/A	N/A	Y	N	N	SA (10^6^), PA non-muc (10^4^), PA muc (10^4^), HI (10^6^)
27	15	2013-10-21	NT	10^6^	S	R	S	S	N	N	N	PA non-muc (10^4^), PA muc (10^6^), SA (10^6^)

^a^Refer to Table 1.

^b^Capsular serotyping done on recoverable isolates.

^c^Antibiotic definitions: Penicillin = PEN, Levofloxacin = LVX, Azithromycin = AZM, Clindamycin = CLI, S = Sensitive, R = Resistant

^d^Influenza vaccine prior to pneumococcus isolation.

^e^Reduced PFT: pulmonary function tests; as defined by a reduction in reduction in FEV_1_ by >10% at time of visit compared to baseline FEV_1_ levels.

^f^Requirement for antibiotics at time of pneumococcus presentation.

^g^PA: *P. aeruginosa,* SA: *S. aureus,* HI: *Haemophilus influenzae,* Bcc: *Burkholderia cepacia* complex, Asp: *Aspergillus.* sp, EC: *E. coli*, CA: *Candida albicans,* Cfc: *Citrobacter freundii* complex, OF: oropharyngeal flora.

^h^N/A: Not available for serotyping or antibiotic susceptibility testing.

^i^NT: non-typeable serotype but with positive *cps* internal PCR product control.

Twelve patients were at a routine clinic visit at the time of sputum collection and subsequent Pneumococcus isolation. Samples from three patients were taken at time of hospitalization or during self-reported acute reductions in health. Only two patients had chest imaging done at time of sputum collection, and both showed pulmonary infiltrates. Six patients had recurrent Pneumococcus isolation, which was discontinuous over a period of time ranging for several months, although none developed chronic infection. One patient had two separate infection periods where different strains of Pneumococcus were cultured a decade apart. Of note, 10 patients did report flu-like symptoms prior to time of Pneumococcus isolation (ranging from one week to five weeks) that consisted of dyspnea (7/10), lethargy (100%), increased sputum production (100%), hemoptysis (20%), and weight loss (40%) from prior clinic visit. Several of the patients with flu-like symptoms also had confirmed viral titers preceding or at time of *S. pneumoniae* isolation and were obtained during periods of acute exacerbation (patient 2 with influenza A, patient 3 with influenza B and patient 10 with influenza A and parainfluenza). No difference was observed in the requirement for acute antibacterial therapies during the clinics from which Pneumococcus was isolated to either those visits occurring in either the year prior to its isolation [10/27 (37%) vs. 14/45 (31%), relative risk (RR) 1.19 (CI 0.62-2.30), p = 0.62] or following [10/27 (37%) vs. 21/45(46%), RR 0.8 (0.44-1.41), p = 0.47].

Of the 27 isolates, 18 were recoverable from frozen stocks and their serotype confirmed (Table 2). Capsular serotypes were performed on the 18 isolates with no predominance observed, rather a distribution of ten different serotypes. Interestingly, three isolates could not be serotyped using multiplex PCR for the thirty most common Pneumococcus serotypes [7] but did display the internal *cps* control confirming Pneumococcus identification. Among the patients with repeated isolates of Pneumococcus, the serotypes identified were not homogenous and were varied even within the same patient. Two patients (8 and 11) had the same Pneumococcus serotype of 23 F over a one month period and 23B over a three month period. Despite extensive chart review, Pneumococcus vaccination records could only be found for one patient (9) with a capsular serotype (23A) that was not included with the 23-valent Pneumococcus vaccine.

Detailed chart review observed that only two isolates were resistant to penicillin upon original testing (7.7%). Amongst the 18 recoverable isolates, additional antibiotic susceptibility testing was performed (Table 2). Four strains (22%) were resistant to azithromycin, but none to clindamycin nor was resistance inducible. Four strains were resistant to levofloxacin (22%), and whilst CLSI breakpoints do not exist for ciprofloxacin, considerably smaller zones of clearance exist suggesting its activity is much more limited (20.5 mm (IQR 18–23) vs 18.5 mm (IQR 14–21), p = 0.07. In addition beta-lactams commonly used in CF with anti-pseudomonal activity including meropenem (47 mm IQR 42–50) and piperacillin-tazobactam (49.5 mm IQR 43–51), showed larger zones of clearing than ceftazidime (34 mm IQR 20–38) although established breakpoints similarly do not exist, p = <0.001. Tobramycin, with no appreciable Gram positive activity beyond Staphylococi, showed very high levels of resistance with zones (<6 mm).

**Figure 1:**
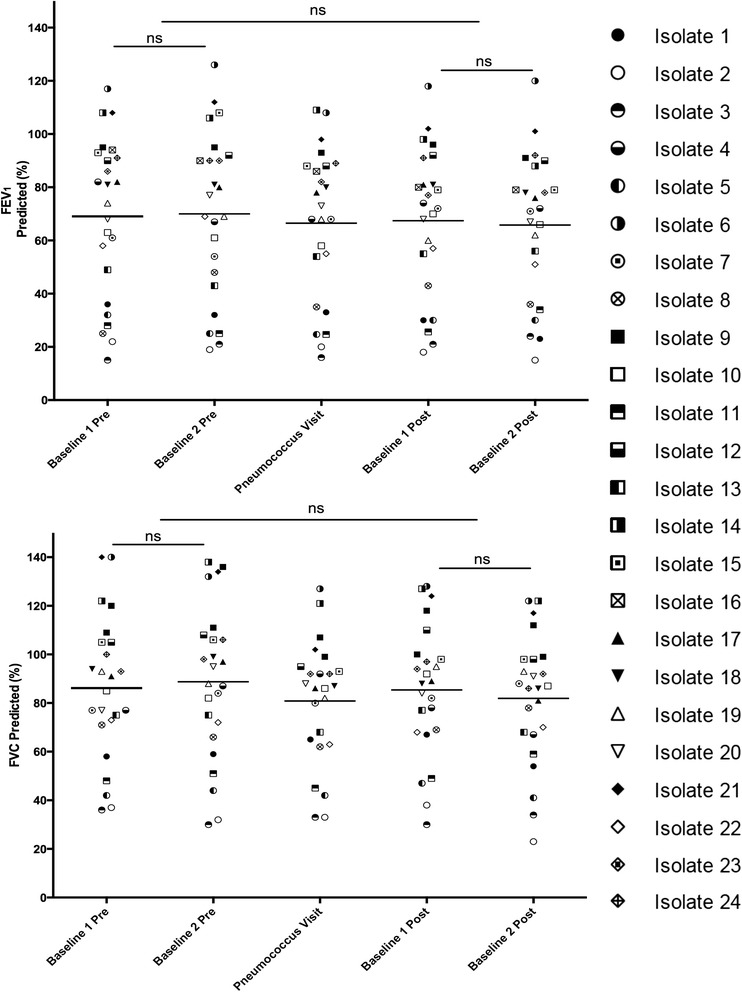
Pulmonary function (FEV_1_ and FVC) among CF patients with Pneumococcus. Spirometery was compared from each patient at each time of Pneumococcal isolation with the values from two clinics immediately preceding and following this visit to assess for impact on lung function. No changes in FEV_1_ were apparent at the time of pneumococcus infection compared to prior (p = 0.6665) or subsequent clinic visits (p = 0.4783). Furthermore, no differences in FVC were observed at time of pneumococcus colonization relative to prior values (p = 0.3354) or follow-up (p = 0.5385). Overall, there appeared to be no significant impact on lung function.

Lung function in the CF patients was compared at time of Pneumococcus isolation with values at the preceding and following two routine clinics to determine if either an acute deterioration or change in disease trajectory occurred as a result of infection (Figure 1). No differences were noted in either percent predicted FEV_1_ or FVC through periods of pneumococcal infection.

## Discussion

Pneumococcus has been well established as a principle respiratory tract pathogen, predominantly in the pediatric and elderly populations. However, CF patients are unique owing to a state of chronic colonization and infection alongside a dysregulated host immune response [9]. Despite this, Pneumococcus is uncommonly reported in CF, and little/no information exists on its prevalence and impact in adult CF patients [2]. Herein we identified that Pneumococcus comprised less than 0.1% of bacterial isolates from a 34-year comprehensive CF biobank where Pneumococcus was identified using classical microbiology protocols, affecting <5% of patients. Furthermore, there were no documented cases of invasive pneumococcal disease, such meningitis and blood stream infection, during this time. The extreme uncommon nature of Pneumococcus in this population was unexpected and may reflect how CF microbiology protocols have been developed and biased to identify classical pathogens such as *S. aureus*, and *P. aeruginosa*.

In CF, the traditional clinical picture has been of chronic lower respiratory tract infections with principal pathogens including *P. aeruginosa, S. aureus,* and *Burkholderia cepacia* complex. Recently, the polymicrobial nature of CF has been demonstrated to comprise of a variety of microbial species that include *Rothia, Prevotella, Klebsiella* and members of the *Streptococcus* genus [5]. While several members of the streptococci, such as *S. anginosus* group, have been implicated in CF disease progression [6], other viridans group streptococci (VGS) including *S. pneumoniae* have not traditionally been considered as primary CF pathogens. However, when specifically sought, using a combination of selective agars such as Mitis–Salivarius agar and high density sampling low levels of Pneumococcus can be identified in up to 10% of sputum samples [2]. In this light it is apparent that traditional approaches to CF sputum culture are likely to be insensitive to the identification of Pneumococcus, particularly at low density.

As a case series and not a controlled study, limited information can be inferred regarding risk factors for Pneumococcus isolation detected by traditional CF protocols. However, 4/15 patients had chronic infection with *H. influenzae, which* is much higher than the standard colonization frequency observed in the adult CF population (<10%) [10]. A high prevalence of smokers similarly was observed, which may explain the disproportionate identification of *H. influenzae*, a common colonizers pathogen in smokers [11]. Furthermore, a large proportion of colonization events were preceded by viral upper respiratory tract infection, which in the general population result in increased colonization potential of these Pneumococcus [12].

The clinical impact of Pneumococcus in CF is poorly understood. We sought specifically to determine if either an acute, or long-term impact followed detection of Pneumococcus from a CF sputum sample using classical CF protocols. We did not identify any statistical difference in lung function at time of isolation compared to the two clinic visits occurring before or after pneumococcal isolation as measured by FEV_1_ and FVC. Furthermore, patients with Pneumococcus in their sputum were no more likely to require antibiotics than during clinic visits occurring in the two years before or after. Finally, no patient went on to develop persistent chronic infection, suggesting that eradication strategies advocated for pathogens such as *P. aeruginosa*, MRSA are not warranted [13,14].

Antibiotic resistance is a growing concern within CF patients, given the longer life expectancy and increased demand on antibiotic use. *S. pneumoniae* is readily competent, allowing horizontal gene transfer between microorganisms for several antibiotic resistance markers including *pbp1a, pbp2, mefE, ermAM* and *tetM* [15]. The presence of *S. pneumoniae* may not be overtly pathogenic in a traditional sense within the CF patient, but may be contributing to the greater antibiotic resistome of the patient by dissemination of antibiotic resistance markers. Antibiotic resistance has been observed in other streptococci species in CF, in particular the *S. anginosus* group, azithromycin (54.9%), erythromycin (52.9%) and clindamycin (49.0%) [16]. While susceptibility data in our study suggests limited resistance to several classes of antibiotics, the Pneumococcal resistance rates have been reported to be on the rise by other groups with 73% to penicillin, 42% to erythromycin, 58% to tetracycline and 67% to trimethoprim-sulfamethoxazole [1]. The discrepancy in Pneumococcus susceptibility values in CF may be attributed to therapeutic management at various sites, global trends of antibiotic use and resistance, and exposure to agents from the pediatric to adult clinics.

Serotyping in this study identified ten different capsular serotypes with no predominant pattern observed. Another work assessing Pneumococcus population structure found the most frequent serotypes in CF were 23 F, 19 F, 6A and 6B [1]. Geographical location and time period may in part affect serotype distribution, as has been described with several antibiotic resistance mechanisms in Pneumococcus [17]. Interestingly, the 23-valent-Pneumococcus vaccine does not cover serotypes 38 and 23A, which were identified in several of our patients. The variety of serotypes within the same patient suggests, given the short time frame between some repeated cultures, likely several strains of pneumococci were present but with only a single isolate recognized as opposed to strain displacement within a single patient. Indeed, co-colonization has been observed with surprising frequency [18]. Coinciding with this, strain displacement of serotypes may be occurring over prolonged periods of time. Furthermore, several isolates could not be serotyped using a strategy to detect the most common capsular serotypes [7], which may suggest acquisition of atypical pneumococci that require further investigation.

The question as to why Pneumococcus was observed in such low prevalence is of particular interest. This may in part be due to in sensitive differentiation from other VGS using standard practice of bile solubility, optochin susceptibility and colony morphology [19]. Furthermore, the use of primarily quantitative microbiology may also result in reduced identification of low quantity organisms within cultures. As well, Pneumococcus colonization is typically higher in young children and then declines with age with a peak in the elderly age group – a group not represented in an adult CF clinic [20]. The median predicted survival of CF patients in Canada is current 50.9 years [21] and as this population ages, Pneumococcal disease may become an issue for older CF patients [22]. More provocatively, however, it may be that chronic infection with classical CF respiratory pathogens and the resultant competition for space and nutrients in the lower airways is protective against extrinsic pathogens such as *S. pneumoniae*.

Current recommendations in CF care involve pneumococcus vaccination with either the 23-valent polysaccharide or, 7 or 13-valent conjugate vaccine [23,24]. The recommendations for these guidelines are derived from those with other respiratory diseases. As such, the need for prevention and treatment of Pneumococcus in CF warrants further investigation as work from this study has shown both low incidence of culture and recovery of patients without further clinical decline, spontaneous clearance and lack of chronic infection. Furthermore, the 23-valent vaccine does not reduce rates of pneumonia but rather is effective in preventing invasive disease such as meningitis and bacteremia [25]. The role of vaccination in CF patients is one that warrants further exploration and perhaps recommendations based on CF specific data as opposed to adaption of recommendations for those with other types of chronic lung disease.

This study has several notable limitations. Few patients were included, and patient data was also limited for those patients who are lost to follow-up or deceased. As we focused on short-term outcomes in this small population we may have been insensitive to longer-term effects. Other clinical parameters may be used in the future to assess if Pneumococcus isolation in CF patients affects disease progression such as number of exacerbations per year, number of hospitalizations, changes in antibiotic regimen and quality of life index scores [26]. As this was a retrospective study evaluating the isolation of *S. pneumoniae* using traditional CF microbiology protocols, it is likely insensitive to the identification of isolates present at low levels. Indeed, prospective studies using semi-selective medias and in depth culturing would be required to determine the prevalence of low level colonization, and how this might impact clinical progression. Furthermore, it may be necessary to adjunct phenotypic culturing with molecular methods as this has been shown to improve efficacy of identification [27].

## Conclusions

In this study, we found Pneumococcus was infrequently identified in the respiratory tract of adults with CF. Furthermore, when Pneumococcus was identified, no apparent adverse impact on outcomes was noted with respect to requirement for rescue antibiotic therapy or changes in lung function.

## Abbreviations

CF: Cystic fibrosis
PCR: Polymerase chain reaction
FEV_1_: Forced expiratory volume in one second
FVC: Forced vital capacity
CBA: Columbia blood agar
CHOC: Chocolate agar
DNA: Deoxyribonucleic acid
CFU: Colony forming unit
IQR: Interquartile range
RR: Relative risk
CLSI: Clinical and Laboratory Standards Institute
mm: Millimeter
NT: Non-typable
N/A: Not applicable
PA: P. aeruginosa
SA: S. aureus
HI: H. influenzae
Bcc: Burkholderia cepacia complex
BMI: Body mass index
PFT: Pulmonary function test
PEN: Penicillin
LVX: Levofloxacin
AZM: Azithromycin
CLI: Clindamycin
